# Thai novice nurses’ lived experiences and perspectives of breastfeeding and human milk in the Neonatal Intensive Care Unit (NICU)

**DOI:** 10.1186/s13006-024-00620-5

**Published:** 2024-03-20

**Authors:** Tippawan Srichalerm, Cynthia S. Jacelon, Lindiwe Sibeko, Jumpee Granger, Carrie-Ellen Briere

**Affiliations:** 1 Ramathibodi School of Nursing, Faculty of Medicine Ramathibodi Hospital, Mahidol University, Bangkok, Thailand; 2 Elaine Marieb College of Nursing, University of Massachusetts Amherst, Amherst, MA USA; 3 School of Public Health & Health Sciences, University of Massachusetts Amherst, Amherst, MA USA; 4 Institute for Nursing Research and Evidence-Based Practice, Connecticut Children’s, Hartford, CT USA

**Keywords:** Breastfeeding, Human milk, Novice nurses, Experiences, Perspectives, NICU, Sick newborns, Preterm infants

## Abstract

**Background:**

Breastfeeding and human milk have well-documented health benefits for newborn infants, particularly those who are sick. However, breastfeeding rates and human milk feeding among infants in neonatal intensive units (NICU) in Thailand are still low; thus, breastfeeding promotion and support are required for Thai mothers of premature infants. Newly graduated nurses can play a critical role within the healthcare support system and can have a significant impact on improving breastfeeding practices in the NICU. The objective of this study was to investigate the lived experiences and perspectives of Thai novice nurses on supporting breastfeeding and human milk feeding in the NICU.

**Methods:**

The study was conducted between March 2021 and May 2022 at three medical centers in the central region of Thailand. This study employed a descriptive phenomenological approach to explore Thai novice nurses’ experiences and perspectives on breastfeeding. Purposive sampling was used to invite Thai novice nurses who have work experience in providing breastfeeding support to NICU mothers and their infants to participate in online interviews using a video conference platform (Zoom). Semi-structured questions were used to interview study participants in their native language. Data were analyzed using Colaizzi’s method of data analysis to identify emergent themes. Member checks, peer debriefing, and self-reflection were applied to ensure the validity and trustworthiness of the study results. Back-translation was also used as a quality and accuracy assurance.

**Results:**

A total of thirteen novice nurses agreed to participate in the study. All were female, and their ages ranged from 21 to 24 years old at the time of the interview. The researchers identified five major themes related to the overall study objectives and research questions. They are: positive attitude toward breastfeeding and human milk, facing breastfeeding challenges at work, self-confidence rooted in experience, professional skill needs, and requiring further support.

**Conclusions:**

Our results suggest that breastfeeding education plays a vital role in encouraging new nurses to provide breastfeeding support to mothers of preterm infants. Establishing breastfeeding support training and innovative learning strategies can be crucial in developing appropriate breastfeeding practice guidelines and policies to support Thai breastfeeding mothers in the NICU.

**Supplementary Information:**

The online version contains supplementary material available at 10.1186/s13006-024-00620-5.

## Background

According to the United Nations Children’s Fund (UNICEF) 2016 global database, Thailand has low rates of breastfeeding for the first six months of an infant’s life [1]. In the preterm population, human milk has been shown to protect against serious newborn conditions, such as necrotizing enterocolitis (NEC) [2–4]. As a result of its known protection in this high-risk population, the use of human milk is presently considered the standard for healthcare in newborn units and the Neonatal Intensive Care Unit (NICU). Data on breastfeeding rates for preterm infants in Thailand are limited.

However, reports by Vermont-Oxford Network and Centers for Disease Control and Prevention (CDC) indicate that in general any human milk rates at discharge from NICU (55.5%) are still below those of healthy infants (76.5%) [5].

During the infant’s NICU stay, it is very challenging for NICU mothers to produce milk due to a variety of factors [6–8]. The factors influencing maternal decision to initiate and then continue breastfeeding can be both emotional and practical, including the conditions of support provided by healthcare professionals [9, 10]. Unless breastfeeding mothers gain assistance from professionals or trained counselors, the mothers who face breastfeeding challenges in the early weeks tend to discontinue breastfeeding and pump breast milk infrequently [11]. Encouragement and assistance with breastfeeding from the nursing staff allow mothers to develop a better understanding of their infants and have the feeling of positively participating in the care of their infants [11]. Some studies revealed that having a perceived support system has a positive influence on the factors associated with the starting of breastfeeding and the length of time to continue it [11, 12].

In regard to the provision of breastfeeding knowledge, adequate breastfeeding education is not always provided to healthcare workers, particularly new nurses, resulting in a lack of several skills that are needed to effectively support breastfeeding mothers [13]. In Thailand, although general breastfeeding education and training programs have been promoted among nursing students and healthcare professionals, the ability of novice nurses to anticipate the complications and challenges of breastfeeding that may occur with mothers of preterm infants is quite limited. In addition to a lack of breastfeeding knowledge and support experience, nurses with less than three years of experience are defined as novice nurses, who require continual verbal and physical cues, and therefore tend not to demonstrate sufficient confidence about safely performing their typical care duties [14, 15]. Their focus on general care activities may in turn reduce the time they can spend learning to effectively help mothers who need support during breastfeeding challenges. Breastfeeding education has been shown to increase nursing confidence and improve the breastfeeding support that nurses provide, thus improving breastfeeding outcomes [16]. Novice nurses are an integral part of the healthcare system that can help encourage and support breastfeeding mothers of preterm infants.

However, there is currently a lack of standardized education and support for Thai novice nurses in this area of maternal and childcare. By improving the education of these nurses in breastfeeding support, these new nurses could make a positive impact on breastfeeding outcomes.

To improve breastfeeding rates for preterm infants in Thailand, healthcare professionals need to understand the perceptions and experiences of novice nurses regarding providing supportive breastfeeding care to parents of their preterm patients. This insight may aid in the development of a system of clinical education that could efficiently help to increase novice nurses’ breastfeeding knowledge and practice. This study explored Thai novice nurses’ experiences and perspectives on breastfeeding in the NICU. The following research questions guided this study: (1) How do novice nurses perceive breastfeeding? (2) To what extent are novice nurses involved with the breastfeeding procedures in the unit? (3) To what extent can the development of breastfeeding support assist with the improvement of novice nurses’ breastfeeding knowledge and skills? Understanding these nurses’ views may inform healthcare professionals to develop clinical education programs to improve nursing practices and skills for the promotion of breastfeeding in sick newborn units.

## Methods

### Study design

This study employed a descriptive phenomenological approach which is a method originally developed by Husserl [17] for collecting, analyzing, and generating data. The objective of phenomenological inquiry is to collect in-depth and multidimensional qualitative information about the attitudes, experiences, feelings, habits, knowledge, and opinions of the participants. In this case, a specific subject area is breastfeeding knowledge, attitudes, and support experiences taking place in NICUs.

Within the method of Husserlian phenomenology, this study followed Colaizzi’s descriptive phenomenological method of data analysis [18]. Colaizzi’s unique seven-step approach ensures a thorough examination, with each stage remaining true to the information. The end product is a succinct and comprehensive explanation of the phenomena under investigation, which has been verified by the people who made it [18].

### Participants and settings

A purposive sampling technique was applied to recruit the study participants. The study participants were from the three medical centers in the central region of Thailand, including Ramathibodi Hospital, Somdech Phra Debaratana Medical Centre (SDMC), Chakri Naruebodindra Medical Institute (CNMI), and Faculty of Medicine Ramathibodi Hospital, Mahidol University. This study included 13 novice nurses who were Thai permanent residents and had zero to three years of work experience in the NICU. By the thirteenth participant, no new insights or themes were produced, and data saturation was reached [19].

### Data collection

Once all approvals were obtained, online advertisements of the study were sent through the head nurses of the three NICUs. The online advertising consisted of email messages, text messages through a cell phone application, and social media outreach (e.g. Facebook Messenger). The online interviews were scheduled at a convenient time for the interviewer and interviewee,, and individual interviews were conducted online over the Zoom program on a secure university platform to ensure security and privacy. Even though the researcher was located in North America and the interviewees were located in Thailand (there is a 12-hour difference), the interviews across time zones were managed. The interviews were conducted in the evenings (6 to 7 p.m.) in Thailand which was a convenient time for all interviewees.

Data collection was conducted through in-depth semi-structured interviews that lasted between 40 and 60 min using a semi-structured interview guide (see Additional file 1). The participants were provided with the study description and the data collection process, including information on how the researcher would summarize their interview and check back with them to ensure that the meaning of their responses was conveyed correctly. The participants were given an opportunity to have their questions answered about the research and/or research process. After providing consent, the participants were video-recorded during their interview using the Zoom program (a video conference or online meeting program). During the interviews, the researcher applied a list of 16 open-ended questions to guide the online discussion. The Zoom interview was done using the Thai language. The interview questions were initially written in English, then translated by the researcher to Thai, and reviewed by another Thai researcher who is an expert in qualitative methods.

The interviews were not limited to the guiding questions; in other words, in addition to the questions listed, prompts were used to extract more elaborate answers if necessary. Participants were encouraged to describe their work experiences using detailed descriptions of their perceptions, attitudes, and beliefs regarding breastfeeding support for NICU families. During each interview, notes were taken to record the expressed feelings and perceptions of the participant, and the participant was asked to check for data accuracy with their experiences at the end of each interview. At the end of each interview, the researcher summarized the main points derived from the interview and asked the participant for validation or elaboration. The interview recording was later transcribed verbatim in the Thai language before interviewing the next participant to ensure the accuracy of that participant’s perspective. In interviews, details may be overlooked, and it can be challenging for the interviewer to remember specific responses to crucial questions. Each note with specific details recorded during the interview was later reviewed by the interviewer when reviewing the interview transcripts.

### Data analysis

The qualitative data of this study was analyzed using Colaizzi’s method [18] with thematic analysis. Colaizzi’s seven-step method was also used to ensure verification of the study findings. In the first step of data analysis, the researcher acquainted herself with the data by reading through all of the participant accounts numerous times. Second, the researcher identified all statements in the interview transcripts for any claims that were directly related to the phenomenon being studied. Third, the researcher derived definitions from the significant statements that are important to the phenomenon. During data gathering and analysis, the researcher also wrote a note to instinctually “bracket” pre-suppositions to remain as close to the phenomena as possible. Fourth, the researcher characterized codes line by line and grouped the concepts discovered into patterns and themes that are shared by all accounts. Fifth, the researcher composed a comprehensive summary of the phenomena that incorporated all of the principal components. Sixth, the researcher simplified the extensive definition into a brief, concise summary that contains only certain facets of the phenomena that are considered important to its design. Finally, the researcher asked some participants if the basic structure statement correctly reflects their understanding.

Language translation was performed after data analysis and obtaining the study results to maintain the original messages and meanings. To safeguard the reliability of the data analysis procedure as well as to corroborate the study findings, the outcomes were reviewed by a different Thai qualitative researcher. Following this, the researcher translated the study findings from Thai to English and had them checked by a Thai bilingual qualitative researcher. The back-translation approach (taking the initial Thai-to-English translation back to Thai and then back again to English) was also conducted to check the accuracy of the study findings and participants’ quotations from Thai to English and English to Thai.

## Results

### Participants’ demographic characteristics

A total of 13 novice nurses agreed to participate in the study which consisted of nine nurses from CNMI, three nurses from SDMC, and one nurse from Ramathibodi hospital. Of the thirteen respondents, all were female, and their ages ranged from 21 to 24 years old at the time of the interview (see Additional file 2).

There were five major themes associated with the research questions regarding Thai novice nurses’ lived experiences and perspectives of breastfeeding and human milk in the NICU. The five main themes presented in the data were as:

Theme 1: Positive attitude toward breastfeeding and human milk;

Theme 2: Facing breastfeeding challenges at work;

Theme 3: Self-confidence rooted in experience;

Theme 4: Professional skill needs; and

Theme 5: Requiring further support.

### Theme 1: Positive attitude toward breastfeeding and human milk

#### Advantages of breastfeeding and human milk

A positive attitude toward breastfeeding in the NICU among novice nurses was related to the appreciation of breastfeeding and human milk. The participants recognized the importance and the positive effects of breastfeeding and human milk for hospitalized infants, specifically premature infants. Most respondents stated that they knew human milk provides essential nutrients for supporting an infant’s immune system. Moreover, in Thailand, the cost of infant formula is expensive and can negatively impact the family budget. Breastfeeding and human milk could save families the cost of preterm formula.

“Personally, breast milk contains immunity. If the mother can pump milk and feed the baby as soon as possible, that would be good because the immunity transferred via the milk will strengthen the baby and build up self-immunity quickly.” (P3).

“…It proves that breast milk contains complete nutrition and is the best choice for the baby growth to become healthy.” (P4).

“Most parents who give birth here are not wealthy. Having much breast milk helps them to save some expenses.” (P2).

“I support breastfeeding as it reduces the expense of milk. There are many expenses to spend. Pumping milk and giving breast milk will relieve this problem. The daily expense of formula milk is high, especially the milk for the preterm infant.” (P9).

#### Negative impacts of not breastfeeding and providing human milk

The participants also described the negative health impacts that occur when a preterm infant did not breastfeed or receive human milk during a NICU stay. Most participants pointed out that human milk significantly improves digestive system health and reduces the risk of necrotizing enterocolitis (NEC) which is a serious gastrointestinal disease affecting premature infants.

“The optimal suggestion for the mother of the preterm infant is breastfeeding. Most NICU infants will have a stomach problem if they don’t get breast milk.” (P10).

“Giving formula milk to the preterm infant will cause more complications, such as intestinal obstruction, etc…” (P13).

“Most NICU infants are ill or preterm babies. Breastfeeding constructs their immunity, and minimizes a chance of NEC.” (P11).

### Theme 2: facing breastfeeding challenges at work

#### Breastfeeding challenges in the NICU: physical and emotional effects

All participants shared their experiences regarding breastfeeding and human milk challenges while working in the NICU. They faced some common breastfeeding problems such as low milk supply and breast engorgement in the NICU mothers. They showed that they were able to solve some breastfeeding problems based on their prior knowledge and experiences. Most participants also indicated that there were differences in emotional reactions between mothers of preterm infants and mothers of healthy infants. The participants said that maternal stress could lead to a low human milk supply. They mentioned that preterm mothers required more time to understand and learn breastfeeding techniques, along with human milk storage than full-term mothers.

“The initial problem of pumping breast milk is there is no milk during the first day of the postpartum period. I would make the understanding to the mother that breast milk might not be produced immediately. Importantly, I will ask her to activate the pumping.” (P3).

“The mother might have breast engorgement sometimes if she doesn’t pump milk completely. We will advise her to do breast compression, breast massage, and pump milk again. In the serious case, we provide a consult at the breastfeeding clinic.” (P5).

“The mother with the premature delivery might not be prepared. She needs time and some advice from us.” (P6).

“As the preterm infant is tiny and can’t be fed from the breast, the mother is stressed and anxious about how to feed the baby. The result of stress is less milk.” (P10).

#### Barriers to continuing breastfeeding and expressing human milk

In addition, most participants were concerned about the barriers to breastfeeding and expressing human milk in the NICU. They highlighted that returning to work had a negative impact on mothers of preterm infants to breastfeed or breast pump. There were some quotes associated with this topic expressed by some respondents.

“Some mothers have to go back to work and don’t want to pump milk or have less time to do so. We think this is also the problem.” (P4).

“Most problems are about returning to work and might not be able to do the pumping. Some feel that they’re uncomfortable doing it every 3 hours and storing the milk. Some don’t have the fridge for storage.” (P5).

### Theme 3: Self-confidence rooted in experience

#### Work challenges with regard to providing breastfeeding information to NICU mothers

Increasing professional confidence and/or self-confidence of novice neonatal nurses was associated with their knowledge and clinical experience. Most participants stated that at the beginning of working in the NICU, they felt stressed, fearful, and had a lack of self-confidence to provide breastfeeding support and other care for NICU mothers and their infants. This stress, fear, and lack of self-confidence were because while they were nursing students, they did not have many experiences providing breastfeeding support to those mothers and their NICU infants.

“Some mothers felt that I was a child who instructed them, and initially, they rarely accepted my information. However, when I was more skilled in providing information based on principles and evidence, those mothers trusted and listened to me more.” (P1).

“In the first few cases of helping latch, I was instructed to try it alone. I tried every way I could but still failed. So, I asked my mentor to help me. When I saw how she did it, I felt like “Oh! I should’ve done this at first.” This case increased my experience, knowledge, and technique for my better understanding at a certain level.” (P13).

“It’s quite difficult to work at the beginning because we haven’t been there before and only observed. When we must do it, it’s challenging. (P12)

“As it is NICU, there is stress and pressure. However, it’s much easier once we went through it.” (P4).

“I’m more confident. I remember that once I wanted to resign because the cases were very serious. I felt it was too much and stressful. But when I overcame it, I enjoy working as it’s very challenging.” (P8).

#### Experiential learning/ training

Some participants shared experiences about when they provided breastfeeding care for the first time as NICU nurses. They said that they learned from their first breastfeeding support experiences and that having work experience and training could build more self-confidence for critical care and breastfeeding support procedures in the NICU. Additionally, some respondents expressed that working in the NICU was challenging and exciting, and they were convinced that the work challenges improved their abilities and work performance. The participants’ quotes are presented below.

“It was very tense. I thought I couldn’t do it. However, when I get used to it, it’s kind of a training to teach me to become a deliberate person.” (P8).

“It’s very stressful when there was a serious case. Once we went through it and faced the same situation again, we have more confidence because we have experienced it before.” (P9).

### Theme 4: Professional skill needs

#### Communication skill needs

There are numerous professional skills needed for new nurses. For this study, the participants explored most skills they need to equip to be confident in their NICU challenges. As far as they were concerned, the most essential skill for NICU nurses to provide effective breastfeeding support was communication skills, specifically interpersonal and persuasion skills. Improving communication skills could boost the chances of breastfeeding success in providing breastfeeding information to support NICU mothers.

“I feel we need more skill as we have to talk. We need speaking skills to convey and make them believe that it’s useful. Talking doesn’t make them recognize the importance as they can’t imagine and don’t want to do it.” (P6).

“It is an explanation! I feel that I say all the key points but they can’t clearly understand some terms. I mean we use some formal terms so the mother doesn’t understand the whole thing. I think giving the example will make them better understand.” (P7).

#### Knowledge sharing and mentoring experience

The study participants also explained that they would look to senior nurses for guidance of breastfeeding and lactation support. They believed that older nurses’ experiences help them develop their work performances and achieve professional goals. Some participants’ quotes related to these statements are provided below.

“I used to read but couldn’t get it. The senior nurse suggests that if we read and have the case at the same time, we will get it quickly. I think the experience helps me to gain more knowledge.” (P2).

“I compared myself with the senior who gave advice to the mother and felt that she made it clear in steps. It might be because they have more experience so I’m very impressed. It’s impressive because she talks smoothly and clearly.” (P5).

### Theme 5: Requiring further support

#### Breastfeeding education materials

The study participants implied that as beginner, novice nurses, they needed support to guide them on the right path to effectively support mothers in breastfeeding in the NICU. The participants mentioned education materials such as clinical nursing guidelines, knowledge booklets/ pamphlets, and knowledge boards that they could access in a breastfeeding room in their unit. Some participants sought breastfeeding information from reliable websites. The knowledge guides provided breastfeeding knowledge regarding the anatomy and physiological basis of breastfeeding, common breastfeeding positions, and human milk storage. Although some knowledge guidelines needed updating, most of them enabled the nurses to review prior knowledge and boost new knowledge before supporting and providing breastfeeding information to NICU mothers.

“For me, the first one is the guidelines of the ward. It’s the pamphlet for the mother that I read and understand before advising the mother. I also search on the internet and select the information from reliable sources.” (P2).

“I have less confident at the very beginning and had to have a pamphlet in hand while explaining to the parent. Though I had been trained, I was afraid to give the wrong and incomplete information that might mislead the mother to the incorrect practice.” (P10).

#### Personal and emotional support

Apart from the material support, the participants also indicated that they required personal and emotional support, namely a mentor and a preceptor, that helped them care for mothers who experienced breastfeeding difficulties and support them in difficult situations. Some participants’ quotes connected to this theme were shown.

“I think I gain knowledge and skills from the workplace where the senior nurses share their experience and information which happened, and what has been taught from the nurse instructors.” (P1)

“…Mostly I learn from the experience and training as it is the real situations which is applicable.” (P13).

“The first source is the senior nurse at the ward. My first time was to listen to the senior nurse who advised the mother. Then, I gained experience constantly.” (P3).

## Discussion

The lived experiences shared in the online interviews presented a rich description of breastfeeding support challenges among Thai novice nurses. The interviews allowed the nurses to reflect and express their feelings and attitudes about breastfeeding support experiences provided to NICU parents.

According to the participants’ responses, most participants had prior knowledge and a positive attitude toward breastfeeding and human milk in the NICU. They recognized that some essential nutrients in human milk improve sick infants’ health and protect against some NICU common conditions. These findings are accordant to the reviewed studies which have shown that human milk contains many nutrients and bioactive factors to develop an infant’s immune system [20]. Human milk could minimize hospitalization costs by reducing the incidence of severe conditions of preterm birth, such as neonatal sepsis and necrotizing enterocolitis (NEC) [21, 22]. Some participants also perceived that breastfeeding and human milk provided a lower financial cost for families compared to the cost of buying formula.

Although knowledge is the main component of nurse preparedness required for breastfeeding support practices, it should be integrated with a positive attitude regarding breastfeeding and human milk in order for nurses to provide strong support for breastfeeding mothers and families [23].

As stated in the participant’s shared experiences, the most common breastfeeding issues in their units included low milk supply and breast engorgement. Research and biological development both support that mothers of preterm infants face more breastfeeding challenges than mothers of healthy infants due to the immature development of the mammary gland at the time of preterm birth [24]. The breastfeeding challenges, namely lower milk supply and the delay in milk production can cause breast symptoms of hardness and swelling (breast engorgement) [25], which is consistent with the participants’ breastfeeding experiences presented in this study.

Other barriers to breastfeeding and human milk provision reported by the participants were returning to work and maternal stress. In the context of Thai culture, although there are 90 days of salary paid for maternity leave by national laws in Thailand, some NICU mothers decide to go back to work during the infant’s NICU stay, and then take maternity leave after their infants are at home. Working mothers often run into the difficulty of balancing breastfeeding and salaried work, which raises the risk of reducing pumping time and thus decreasing their overall milk volume. Going back to work and having lactation support limitations at the workplace have been mentioned as contributing causes to the low breastfeeding rates among working mothers [26]. Regarding maternal stress, the NICU environment and the birth of a high-risk infant can cause stress for breastfeeding mothers. The stress of NICU mothers may become even worse than that of mothers of healthy infants [27]. These mothers may need additional support from NICU staff to help them cope with ongoing NICU challenges. Many of these challenges and barriers can be minimized with effective education and support provided by NICU nurses to preterm mothers [28].

About the participants’ expressions, the participants expressed that they gained more self-confidence to support NICU mothers after having breastfeeding support experience at work. Most participants learned from their mistakes during their initial breastfeeding practices at their unit. Some participants could apply the lessons learned from their experience to support breastfeeding procedures for the next NICU mother they cared for. These results are in line with some studies that have indicated that most new graduates lack confidence when they face a new challenge, and their confidence can be gained by experience [29].

Based on the participants’ statements, improving professional communication skills among new nurses, especially interpersonal and convincing skills, is needed. This study result is consistent with survey evidence which showed that efficient communication was the top three necessary professional skills for new graduate nurses [30]. Effective communication could increase the achievement of breastfeeding support for NICU mothers. Nurses who are equipped with these communication skills could provide clear and confident breastfeeding support to those mothers. On the other hand, some studies suggested that health professionals who lack communication skills face a difficult time approaching their patients [31, 32]. They are more likely to transfer one’s problems to the other [32]. Thus, indisputably, communication skills are one of the professional skills needed and equally critical as clinical skills in new NICU nurses.

The study participants expressed that due to the complexity of the NICU settings, they needed both physical education resources and emotional support to reliably provide breastfeeding information and procedures to NICU mothers and infants. However, previous evidence reported that although there were several support programs provided for new graduate nurses in the first year of clinical practice, the programs might not be able to respond to the nurses’ demands [33]. Without appropriate support, new graduate nurses intend to leave the profession [34]. Providing support depending on learners’ needs tends to be one of the personal development strategies to improve learning capacity and promote professional practices among new nurses.

Whenever this type of research is done, it is important to recognize there can be a response bias where participants alter their responses based on their perception of what the interviewer wants to learn. In this study, the researcher was concerned about the response bias and reduced the bias by building rapport with the participants, asking neutral questions, asking additional questions to confirm the participants’ accurate answers, and avoiding a leading answer to the participants. Reducing response bias allows the researcher to obtain the actual results which are valuable information for implementing learning strategies to help improve novice nurses’ breastfeeding knowledge and skills.

Due to the health risk associated with feeding immature NICU infants, and the benefit of human milk for infant health, breastfeeding rates and human milk receipt in the NICU need to be improved. In the meantime, we focused on the practitioner’s ability to support NICU parents. In the next step, we will look at incorporating mothers of preterm infants in Thailand and exploring their needs for breastfeeding support during their infants’ NICU stay. Another recommendation for future research involves considering alternative methods to evaluate other barriers to improving breastfeeding initiation and continuation rates in Thailand. Further studies would focus on establishing breastfeeding education programs and specific breastfeeding training based on the interests of the new nurses, leading to the improvement of learners’ motivation and engagement.

## Conclusion

This study explored Thai novice nurses’ lived experiences and perspectives toward breastfeeding and human milk in the NICU. The study sought to understand new nurses’ attitudes regarding facilitators and barriers to breastfeeding support for mothers of sick infants in Thailand. Study findings support the need for further research to initiate breastfeeding support training programs and innovative learning strategies for enhancing breastfeeding knowledge and skills among new graduate nurses, to improve the quality of the breastfeeding support system in Thailand.

Our findings show that in our sample of new NICU nurses in Thailand, many have positive attitudes toward breastfeeding and human milk. The new nurses are more likely to learn more and stay interested in learning situations [35]. Thus, adding more practical breastfeeding education may improve self-confidence and clinical skills among novice nurses. Based on the results around skills and self-confidence, we suggest the incorporation of breastfeeding simulation and practice with role-playing into the nursing curriculum. Besides, as clinicians, we realize Benner’s novice to expert model of skill acquisition to assess NICU nurses’ abilities and what support they need to move to the next level of confidence [14]. We also want to review and revise breastfeeding support guidelines and other knowledge materials to help new nurses improve their clinical practice and gain self-confidence, to improve clinical outcomes.

Furthermore, understanding novice nurses’ experiences and perspectives could enable academic nurse educators, nurse managers, and senior staff nurses to realize new graduate nurses’ breastfeeding knowledge, skills, and support demands. Supporting and providing appropriate breastfeeding support training based on the nurses’ needs would motivate the nurses to learn effectively. It can be concluded that the study findings can be a piece of meaningful information for the first step in developing breastfeeding education for nurses in Thailand. Another step would aim to track the improvement of breastfeeding support programs and training following the learners’ needs, resulting in enhancing the quality of breastfeeding support in Thailand.

### Limitations and considerations

Data collection through online interviews was an extremely challenging task. In the report of Krouwel et al. (2019), in-person interviews provided more words and statements than video call interviews [36]. During in-person interviews, the researcher can take more time to build rapport with the research participants and can notice participants’ responses. Online interviews might limit some activities to encourage the participants’ elaboration. Therefore, during the online interviews, some information may not be mentioned by the participants. Furthermore, even though the Zoom program provides an extensive range of visual and verbal exchanges [37], the observation of participants’ non-verbal signals may be limited. The lack of some nonverbal behaviors, including posture and some body movements, could reduce the substantial information in support of the meaning of the participants’ lived experiences and perspectives. Nonetheless, online interviews over the Zoom program can be video recorded which assisted the researcher in reviewing and obtaining more detailed and precise qualitative information.

Another study limitation was related to the study participants. Since participants were willing and interested to be part of this study and had generally positive breastfeeding attitudes, this finding of positive attitude may not be representative of the entire population of Thai novice nurses. If novice nurses’ attitudes are not positive, several factors influencing breastfeeding attitudes should be investigated. Moreover, according to the demographic information, the participants were from two nursing schools and three medical centers located in the middle region of Thailand. They had the experience of breastfeeding support in the NICU in only one region of Thailand which consists of six regions in total. Their breastfeeding support experiences may be limited because they may not face a variety of breastfeeding challenges on account of the similar ethnic backgrounds, and educational levels of NICU mothers in the region where they worked.

### Electronic supplementary material

Below is the link to the electronic supplementary material.

Supplementary Material 1 Additional file 1: The semi–structured interview guide

Supplementary Material 2 Additional file 2: The demographic characteristics of participants

## Data Availability

The dataset generated and/or analyzed during the current study are not publicly available due to Thai native participants but are available from the author on reasonable request.
