# Supplementary material for: Thai novice nurses’ lived experiences and perspectives of breastfeeding and human milk in the Neonatal Intensive Care Unit (NICU)
Source: Int Breastfeed J. 2024 Mar 20;19:20. doi: 10.1186/s13006-024-00620-5 (PMC10956329; doi:10.1186/s13006-024-00620-5)
Supplement: Supplementary file 1 — Supplementary Material 1 Additional file 1: The semi–structured interview guide [file 13006_2024_620_MOESM1_ESM.pdf]

The Demographic Characteristics of Participants (n=13)

Participant No.	Age (year old)	Work Experience (Year)	Workplace/ Nursing School
1	22	1-2	CNMI/ Ramathibodi
2	22	1-2	CNMI/ Burapha
3	24	2-3	CNMI/ Ramathibodi
4	21	0-1	CNMI/ Ramathibodi
5	21	0-1	CNMI/ Ramathibodi
6	23	2-3	CNMI/ Ramathibodi
7	21	0-1	CNMI/ Ramathibodi
8	23	2-3	CNMI/ Ramathibodi
9	21	0-1	CNMI/ Ramathibodi
10	22	0-1	RAMA/ Burapha
11	22	1-2	SDMC/ Ramathibodi
12	21	0-1	SDMC/ Ramathibodi
13	21	0-1	SDMC/ Ramathibodi
