# Supplementary material for: Thai novice nurses’ lived experiences and perspectives of breastfeeding and human milk in the Neonatal Intensive Care Unit (NICU)
Source: Int Breastfeed J. 2024 Mar 20;19:20. doi: 10.1186/s13006-024-00620-5 (PMC10956329; doi:10.1186/s13006-024-00620-5)
Supplement: Supplementary file 2 — Supplementary Material 2 Additional file 2: The demographic characteristics of participants [file 13006_2024_620_MOESM2_ESM.pdf]

**Semi-structured interview guide**

| English | Thai |
|---|---|
| Why did you decide to be a neonatal nurse? | ทำไมคุณถึงตัดสินใจมาเป็นพยาบาลทารกแรกเกิด |
| How long have you been working as a neonatal nurse? | คุณทำงานมานานเท่าไหร่ |
| Please tell me more about your work experiences, such as your duties, roles, or responsibilities (How do you perceive in the NICU?) | ช่วยเล่าถึงประสบการณ์การทำงานของคุณ หน้าที่ที่รับผิดชอบ/ คุณมีรู้สึกอย่างไรในการทำงานที่นี่ (NICU) |
| What do you think about breastfeeding in the NICU? (Attitude) | คุณมีความคิดเห็นอย่างไรเกี่ยวกับนมแม่ใน NICU |
| Why is breastfeeding so important/not important in the sick newborn unit? (Knowledge & Attitude) (How do your breastfeeding support actions support or not?) | คุณคิดว่านมแม่มีความสำคัญหรือไม่<br>ทำไมคุณถึงคิดว่านมแม่สำคัญ/ ไม่สำคัญกับเด็กป่วยใน NICU |
| Before working as a nurse, did you take any courses about breastfeeding support? (Please tell me more about the course/ if not, do you want to take any breastfeeding support training courses?) | ก่อนการทำงาน คุณมีโอกาสได้เข้าเรียน และ/ หรือฝึกปฏิบัติเกี่ยวกับการส่งเสริมนมแม่ในหอผู้ป่วยหรือไม่ เล่าประสบการณ์การเรียน และ/หรือการฝึกปฏิบัตินั้น ๆ |
| If your friend gave preterm birth and asked your advice about breastfeeding or breast pumping, what is your suggestion? (knowledge) | ถ้าเพื่อนคุณมีบุตรที่เกิดก่อนกำหนด แล้วถามคุณเกี่ยวกับการให้นมแม่ และการปั๊มนม คุณจะให้คำแนะนำอย่างไร |
| If your friend had some breastfeeding problems, what should you do or what is your advice? (what breastfeeding challenges did you face before?) (experiences) | ถ้าเพื่อนคุณมีปัญหาเกี่ยวกับน้ำนม เช่นน้ำนมน้อย หรือปัญหาเกี่ยวกับเต้านม เช่นปวดเต้านมมาก คลำได้เป็นก้อน คุณจะให้คำแนะนำอย่างไร |
| If you can think of the most beneficial breastfeeding help, support, or advice you have had with breastfeeding, who/what would be the source? | คุณคิดว่าสิ่งที่ช่วยคุณให้มีความรู้ และทักษะในการส่งเสริม สนับสนุน ให้คำแนะนำเกี่ยวกับนมแม่ได้**มาก**ที่สุดคืออะไร หรือใคร |
| Why was this person/source the most helpful for you? | ทำไมคุณถึงคิดว่าบุคคลหรือแหล่งความรู้นั้นช่วยคุณได้มากที่สุด |
| What made it a positive experience? | คุณเคยมีโอกาสได้เรียนรู้ประสบการณ์ที่ดีแบบนั้นหรือไม่ อย่างไร |
| If you can think of the least helpful (or the most negative) breastfeeding help, support, or advice you have had with breastfeeding, who/what would be the source? | คุณคิดว่าสิ่งที่ช่วยคุณให้มีความรู้ และทักษะในการส่งเสริม สนับสนุน ให้คำแนะนำเกี่ยวกับนมแม่ได้**น้อย**ที่สุดคืออะไร หรือใคร |
| Why was this person/source the least helpful for you? | ทำไมคุณถึงคิดว่าบุคคลหรือแหล่งความรู้นั้นช่วยคุณได้น้อยที่สุด |
| What made it a negative experience? | คุณเคยมีโอกาสได้เรียนรู้ประสบการณ์ที่ไม่ดีแบบนั้นหรือไม่ อย่างไร |
| Is there anything else you would like to share about your experiences of breastfeeding support? | คุณอยากเล่าประสบการณ์เพิ่มเติมหรือไม่ |
| What do you want to tell me before leaving? (in the end of the interview) | มีอะไรที่คุณต้องการถาม หรือบอกก่อนจะจบการสนทนาหรือไม่ |
